# An Update on Interleukin-9: From Its Cellular Source and Signal Transduction to Its Role in Immunopathogenesis

**DOI:** 10.3390/ijms20092113

**Published:** 2019-04-29

**Authors:** Sushmita Chakraborty, Katharina F. Kubatzky, Dipendra Kumar Mitra

**Affiliations:** 1 Department of Transplant Immunology and Immunogenetics, All India Institute of Medical Sciences, New Delhi 1100029, India; 2 Zentrum für Infektiologie, Medizinische Mikrobiologie und Hygiene, Universitätsklinikum Heidelberg, Im Neuenheimer Feld 324, 69120 Heidelberg, Germany; kubatzky@uni-heidelberg.de

**Keywords:** interleukin-9, inflammatory diseases, signal transduction

## Abstract

Interleukin-9 (IL-9) is a pleiotropic cytokine and was primarily studied in the context of T helper 2 (T_H_2)-associated immuno-pathological conditions such as asthma and parasitic infections. There was a paradigm shift in the biology of IL-9 after the recent discovery of T_H_9 cells, a new subtype of T_H_ cells which secrete IL-9 in copious amounts. This has resulted in renewed interest in this cytokine, which was neglected since discovery because it was considered it to be just another T_H_2 cytokine. Recent studies have shown that it has multiple cellular sources and is critically involved in the immune-pathogenesis of inflammatory diseases and in guarding immune tolerance. In this review, we will discuss its discovery, gene organization, cellular sources, and signaling pathways. Especially, we will give an update on the recent development regarding its relevance in the immune pathogenesis of human diseases.

## 1. Introduction

In 1988, Interleukin-9 (IL-9) was first discovered and reported by Jacques van Snick’s laboratory. Using Helper T (T_H_) cell lines that were able to proliferate without antigen specific stimulation, they observed that some cell lines were able to secrete a factor which supported long-term growth [1]. Gel filtration resulted in the purification of a protein with a molecular weight between 32–39 kDa. The factor was therefore designated as P40; however, when the factor was cloned, it was found that the actual protein size is only around 14 kDa. The discrepancy was attributed to heavy N-linked glycosylation of the cytokine. Experiments using neutralizing antibodies against IL-2 and of IL-4 indicated that the effect of P40 on T cell growth was direct and not mediated through either IL-2 or IL-4 [1]. Around the same time, another factor was characterized from long term T Helper 2 (T_H_2) cell lines and was termed T Cell Growth Factor III (TCGF III) [2]. Comparative sequence analysis revealed that TCGF III was identical with P40 but not with any other known cytokines or colony stimulating factors [3]. Today, TCGF is known as IL-2, and TCGF II is known as IL-4. Apart from T cell clones, naive murine CD4 T cells were also observed to secrete this factor P40 [4]. At a similar time, a novel factor Mast Cell Growth Enhancing Activity (MEA) was identified by researchers from the Institute for Experimental Haematology, München, to which mast cell lines responded. The effect of the factor MEA was distinct from other known mast cell growth factors such as IL-3 or IL-4 [5,6]. Finally, in 1990, it was clear among researchers that TCGF III and MEA both represented the factor P40 [7]. In the following years, different research groups were apprehensive about its designation as TCGF III or P40 or MEA and, therefore, preferred designating it as IL-9 due to the pleiotropic function of the cytokine [8]. IL-9 is now known to support the growth of not only mast cells and T cells but also other cell types such as erythroid progenitors, fetal thymocytes, human megakaryoblastic leukemic cell lines, and myeloid precursors [9,10,11,12].

Though this cytokine has been known for three decades, for a long time IL-9 was mainly studied in the context of T_H_2-associated immuno-pathological conditions, since IL-9 played a role in asthma, IgE class switch recombination, and the resolution of parasitic infections. More recently, IL-9 has attracted renewed interest owing to its involvement in the immune-pathogenesis of inflammatory diseases, its association with different T_H_ cell types, and its less studied role in immune tolerance. In this review, we discuss its cellular sources, IL-9 mediated signaling, and its role in immune pathogenesis.

## 2. IL-9 and Its Receptor

The molecular characterization of the mouse *Il9* gene revealed that the gene is located on chromosome 13, whereas its human homologue is located on chromosome 5 within the T_H_2 cytokine cluster (IL-2, IL-4, GM-CSF, and IL-13) in the region q31–35 [13,14]. A very similar genomic organization is observed between human and mouse genes, consisting of five exons and four introns. 63% similarity is also observed in the three untranslated regions of human and mouse *Il9*. The mouse IL-9 peptide is a basic single-chain glycoprotein consisting of 126 amino acids and folds into a four-α-helix bundle like other cytokines from the IL-2 family. The peptide is synthesized as a precursor consisting of 144 amino acids including an 18 amino acid signal sequence peptide [4,15]. The mature mouse protein shares 55% homology with human IL-9 with a perfect conservation of 10 cysteine residues which are essential for the formation of disulphide bonds. Despite the overall similarity between human and mouse IL-9, only murine IL-9 is active on human cells, while human IL-9 fails to show any effect on murine cells. 

In 1990, Jacques van Snick’s laboratory first demonstrated that the IL-9 receptor alpha (IL-9Rα), a member of type I hematopoietin receptor superfamily, has high affinity (Kd of approximately 100 pM) for IL-9. The expression of this 64-kDa glycoprotein is reported on a variety of hematopoietic cells [16]. Similar to the other members of the IL-2 receptor family, IL-9Rα also forms a heterotypic receptor complex with the common gamma (γc) chain. In the IL-9R heterocomplex, the IL-9Rα chain is the ligand binding domain and γ chain serves as the signaling subunit [17,18,19]. The mouse IL-9Rα comprises of 468 amino acids; whereas its human homologue consists of 521 amino acids. The IL-9Rα subunit is characterized by four extracellular cysteines and the conserved WSXWS motif, while the intracellular domain contains BOX1 consensus sequence and a serine rich region. IL-9Rα is found in both membrane bound and soluble forms, whereas the γc subunit is observed only in a membrane bound form. In the absence of IL-9, 25% of the IL-9Rα associates with the γc domain, but, in the presence of IL-9, the percentage of heterotypic receptor complexes increases [20]. 

## 3. IL-9 Receptor Signaling

IL-9 binding to the ligand-binding subunit IL-9Rα results in the formation the IL-9R heterocomplex. A hallmark of the IL-9R heterocomplex is the absence of any intracellular enzymatic activity, and, therefore, Janus kinases (JAK) need to mediate the phosphorylation of the receptor [21]. Upon IL-9 binding to the receptor, a conformational change occurs in the IL-9R heterocomplex, which allows JAK molecules to bind to the proline rich BOX1 motif in the membrane-proximal region of IL-9Rα. It has been observed that deletion or truncation of this BOX1 motif completely abolishes IL-9-induced phosphorylation of tyrosine residues of JAK1 and JAK3, suggesting that the BOX1 motif in IL-9Rα plays a prominent role in the IL-9-induced activation of JAK kinases [22]. JAK1 associates with IL-9Rα, whereas JAK3 binds to γc. Phosphorylated JAK1 and JAK3 then mediate the phosphorylation of receptor tyrosine residues. Phosphorylated tyrosine residues act as docking sites for the downstream Src homology 2 (SH2) domain containing signaling molecules such as Signal Transducer and Activator of Transcription (STAT) transcription factors, insulin receptor substrate (IRS), and the adaptors of the Mitogen-Activated Protein Kinase (MAPK) pathways. 

Among the five cytoplasmic tyrosine residues in IL-9Rα, the IL-9 mediated activation of STATs (STAT1, STAT3, and STAT5) depends on a single phosphorylated tyrosine residue (tyrosine 367) [23]. Distinct amino acids near tyrosine 367 are critical in the activation of specific STAT proteins of the STAT family: Proline 369 and glutamine 370 are required for full STAT1 activation; glutamine 370 is required for STAT3; and leucine 368 is required for STAT5 [23]. STAT proteins contain a putative SH3 domain, and it is possible that a proline-rich region between 121 to 134 in the cytoplasmic tail of the IL-9Rα stabilizes the interaction of the receptor with the STAT protein and supports efficient signal transduction [24]. Subsequently, homodimers of STAT-1, STAT-3, and STAT-5, as well as STAT heterodimers (STAT-1/STAT-3), translocate to the nucleus, where they bind to regulatory sequences and initiate de novo gene expression regulating cellular functions. Glucocorticoids are the potent immune-suppressive agents, which reduce the expression of inflammatory cytokines by inhibiting transcription factors like Activation Protein (AP-1) and NFkB. Glucocorticoids have also been observed to block IL-2 signaling by inhibiting STAT5 activation in primary T cells [25]. Similar inhibition of signaling by glucocorticoids has been observed in other members of the IL-2 receptor family [25]. Thus, the IL-9 mediated activation of JAK-STAT pathway can be inhibited with glucocorticoids. 

In various hematopoietic cells, IL-9 also activates insulin receptor substrates (IRS) 1 and 2 [26,27]. These proteins are large molecules which contain a protein tyrosine binding (PTB) domain, a pleckstrin homology (PH) domain, and many phosphorylation sites for serine/threonine and tyrosine residue. Yin et al. have shown that, following IL-9 stimulation, JAK1 associates with IRS-1 directly; however, they failed to observe a similar association of JAK3 with IRS-1 [27]. In addition, the sequence (PL-X4-NPXYXSXSD), which is conserved among IL-4, insulin, and an insulin-like growth factor for the interaction with IRS-1, is not present in the IL-9Rα subunit. Following JAK mediated phosphorylation; IRS proteins interact with other SH2-containing signaling proteins, such as the regulatory subunit of Phosphatidylinositol-3 Kinase (PI3-K) p85, causing the activation of the PI3-K catalytic subunit p110 [28]. The PI3-K then activates downstream signaling molecules like PI3-K-dependent kinase (PDK) and Akt. Akt then phosphorylates BAD and protects cells by preventing caspase-mediated apoptosis.

IL-9 also activates the MAPK pathway in several cell lines of lymphoid and hematopoietic origin, but the IL-9 mediated MAPK activation is weak compared to other cytokines like IL-3 [29]. Following IL-9 binding to the IL-9Rα, the SHC is phosphorylated, which then binds to Grb2. Grb2 subsequently activates Son of Sevenless (SOS), a GTP exchange factor for Ras GTPases, resulting in the activation of Ras GTPase. Ras GTPases then activate Raf kinase, followed by the activation of MEK1/2 and ERK1/2. It is not clear how SHC binds to IL-9Rα as the IL-9R lacks any know binding site for SHC mediated ERK1/2 activation, indicating the presence of additional adaptor protein between IL-9Rα and SHC. IRS might bind to SHC in IL-9-induced signaling pathway, but the IRS activated by IL-9 did not seem sufficient for inducing MAPK activation [30]. 

There are protein families which play a critical role in the regulation of duration and termination of cytokine receptor signaling events. These include Suppressors of Cytokine Signaling (SOCS), Protein Inhibitors of Activated STATs (PIAS), and the SH2-containing phosphatase SH-PTP2 [31]. SOCS stops the receptor activation by blocking the activation of STATs. IL-9 induces the expression of three members of the SOCS family: Cytokine-Inducible SH2-containing Protein (CIS), SOCS-2, and SOCS-3. However, only the overexpression of SOCS-3 resulted in inhibition of IL-9-induced signal transduction [32]. Unlike SOCS, PIAS proteins are constitutively expressed. PIAS bind to dimers of activated STATs in the IL-9 signaling pathway and prohibit binding of STATs to specific DNA sequences in the nucleus. In IL-9 signaling, phosphatases only play a minor role in IL-9R deactivation, as the treatment with a phosphatase inhibitor did not show any effect on the phosphorylation level of the receptor [33].

**Figure 1:**
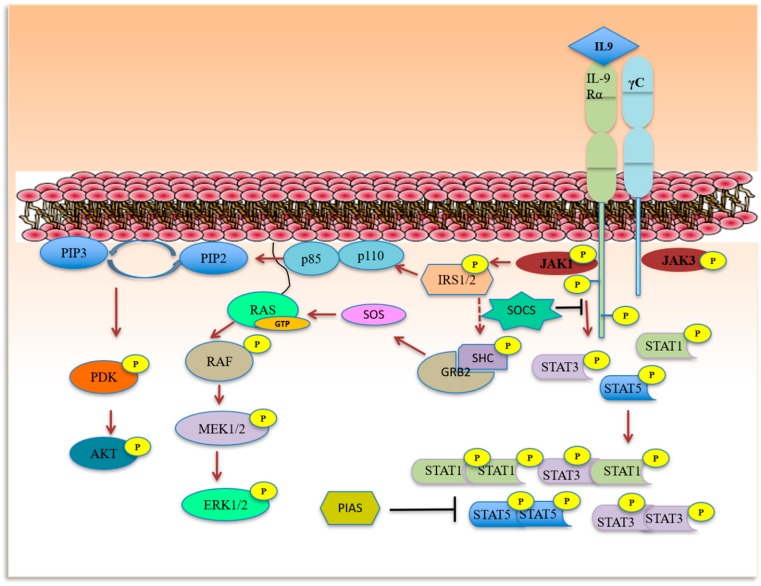
IL-9 mediated signal transduction. IL-9 binding results in the formation the IL-9R heterocomplex, which induces phosphorylation of JAKs. Phosphorylated JAKs then activate signaling intermediates of JAK-STAT, IRS—PI3 kinase, and MAPK pathways. (STAT—Signal Transducer and Activator of Transcription; IRS—insulin receptor substrate; MAPK—Mitogen-Activated Protein Kinase; PI3-K—Phosphatidylinositol-3 Kinase having two subunits, p110 and p85; PDK—PI3-K dependent kinase; PIAS—Protein Inhibitors of Activated STATs; SOCS—Suppressors Of Cytokine Signaling).

Another important negative regulatory pathway is the down-regulation of cell surface receptors, which prevents IL-9 molecules from binding to the receptor. After IL-9 stimulation, downregulation of cell surface IL-9R occurs partly through the Ubiquitin-proteasome pathway. The IL-9R was shown to undergo polyubiquitination upon stimulation with IL-9, and the ubiquitinylated receptors were then targeted to proteasomal degradation through association with the Vasolin-Containing Protein (VCP), a proteasome-associated putative chaperone [34]. The IL-9 mediated signaling is summarized in Figure 1. 

## 4. Cellular Sources of IL-9

Since its discovery in 1988, T cells have been considered as the major source of IL-9. Initially, they were associated with a T_H_2 phenotype owing to observations like its gene positioning within the T_H_2 cytokine cluster, its preferential secretion with other T_H_2 cytokines, and the increase in its expression in the T_H_2-prone BALB/c mouse strain during *Leishmania major* infection but not in the T_H_1-prone C57BL/6 mouse strain [35,36]. It was also observed that treatment of BALB/c mice with a neutralizing antibody against IL-4, a key mediator of the T_H_2 type, could suppress IL-9 synthesis and a correlation of IL-9 production with the proliferation of antigen specific T_H_2 cells in BALB/c mice that were detected after four weeks of infection, suggesting its association with a T_H_2 phenotype [35]. In 2008, two papers provided evidence that a distinct subset of CD4+ cells exists which predominantly secretes IL-9 and does not express any other T_H_ cell lineage-specific cytokine or transcription factor. These cells were accordingly termed T_H_9 cells. These papers suggested that TGF-β, in the presence of IL-4, reprograms CD4+ T cells into T_H_9 cells [37,38]. It was also shown that IL-9 secretion by murine T_H_2 cells was strongly dependent on exogenous TGF-β, and that TGF-β could redirect committed T_H_2 cells towards a T_H_9 phenotype [38]. The search for a T_H_9 specific transcription factor revealed the key involvement of Interferon-Regulatory Factor 4 (IRF4), Basic Leucine Zipper Transcription Factor ATF-like (BATF), and PU.1 [39]. Accordingly, ectopic expression of PU.1 in either T_H_2 cells or T_H_9 cells increased IL-9 production, suggesting that PU.1 is capable of inducing IL-9 production in T_H_ cell subsets [40].

Apart from the T_H_9 and T_H_2 subsets, purified ex vivo and in vitro generated mouse T_H_17 cells produce IL-9 [41]. Multiple Sclerosis (MS), which is a T_H_17 driven disease, neutralizing IL-9 or IL-9R knockout attenuates disease progression and severity in animal model of MS [41]. The amelioration of the disease status correlated with a decrease in the number of T_H_17 cells, implicating a significant contribution of IL-9 in T_H_17-mediated inflammatory diseases. IL-9 produced by T_H_17 cells acts with TGF-β to differentiate naïve CD4+ T cells into T_H_17 cells and to further amplify the T_H_17 subset. In addition, the frequency of T_H_17 cells induced under T_H_17 polarizing conditions in vitro was significantly reduced in IL-9R knock out T cells compared to wild type CD4+ T cells [42]. In response to T_H_17 polarizing conditions, human CD4 T cells secrete IL-9 but fail to co-express IL-17 and IL-9. However, these CD4 cells can co-express both cytokines (IL-17 and IL-9) under T_H_17 inducing conditions after repeated stimulation [43]. TGF-β also induces IL-9 expression in memory CD4 T cells [43]. The addition of TGF-β to the T_H_17- memory cell inducing cytokines (IL-1 β, IL-6, IL-21, IL-23) results in the marked co-expression of IL-9 in IL-17 producing memory CD4 cells. Furthermore, in autoimmune diabetes, a higher frequency of memory CD4 cells co-expressing IL-9 and IL-17 has been observed, indicating their role in autoimmune diseases [43]. 

Contradictory reports are available regarding the expression of IL-9 from regulatory T cells (Tregs) [44]. In an animal model of skin allograft and nephrotoxic serum nephritis, Tregs mediated allograft tolerance, and nephroprotective effects were observed to be mediated through IL-9 [45]. IL-9 neutralizing reversed the immune suppressive effect of Tregs in these mouse models. However, Treg cells from FoxP3.GFP reporter mice did not express IL-9 at the gene and protein level [42]. In addition, naïve CD4+ T cells converted into iTregs in the presence of TGF-β did not produce IL-9. Apart from helper T cells, cytotoxic CD8+T cells (T_C_) can differentiate into IL-9-producing cytotoxic CD8+T cells (Tc9) cells under T_H_9 polarizing conditions [46].

**Figure 2:**
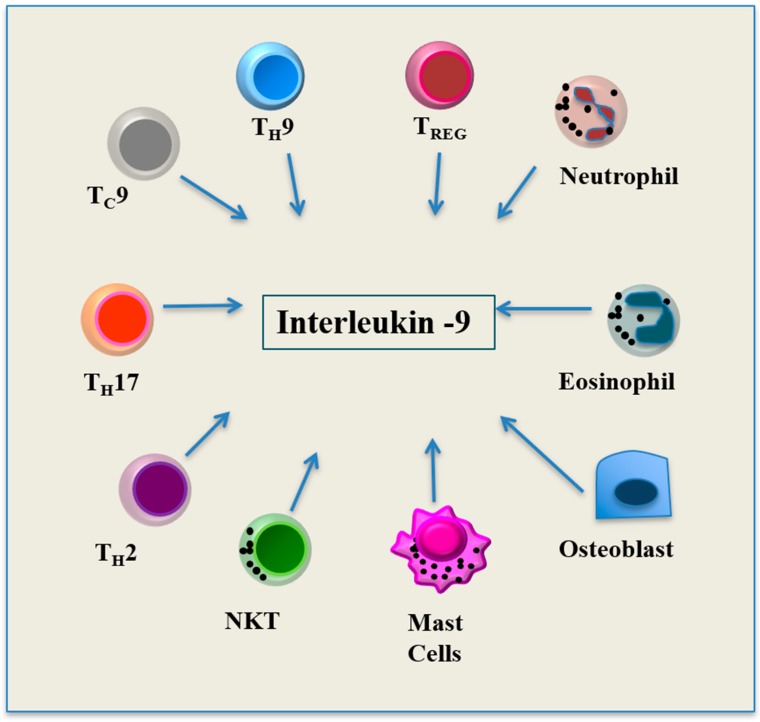
Various cellular sources of IL-9.

Other immune cells have also been observed to secrete IL-9. Mucosal mast cells profusely secrete IL-9 and are critical in driving mastocytosis [47]. In asthmatic airways, mast cells are an important source of IL-9. In addition, human eosinophils and neutrophils have been observed to secrete IL-9 [48,49]. Innate lymphoid cells which are an important component of the innate immune system have also been observed to secrete IL-9 [50]. Mouse Natural Killer T (NKT) cells produce IL-9 upon stimulation with IL-2 [51]. The deficiency of NKT cells in a mouse model of allergic airway inflammation correlated with a decreased expression of IL-9 in lungs, suggesting the involvement of NKT-mediated IL-9 secretion in inflammation [52]. NKT cells that have undergone transformation to nasal NKT cell lymphoma cell lines also produce IL-9 [53]. Large numbers of IL-9 secreting NKT cells are observed in the histological sections of lymphomas from patients with nasal NKT cell lymphomas [53]. Osteoblasts also produce IL-9, which helps in supporting osteoblast mediated megakaryopoisis [54]. Thus, it is very evident from these observations that IL-9 has multiple cellular sources (Figure 2) which might influence its pleiotropic functions.

## 5. Role of IL-9 in Inflammatory Condition and Immune Tolerance

Recent observations in animal models and patient samples have revealed a role for IL-9 in various inflammatory conditions which are discussed below. 

### 5.1. Airway and Allergic Inflammation

In asthmatic patients, increased levels of IL-9 are observed in the serum, lung, and sputum [55,56,57,58,59]. Along with IL-9, the expression of its receptor (IL-9R) is also elevated in the lungs of asthmatic patients. In transgenic mice, the overexpression of IL-9 in the lungs resulted in hypertrophy of the airway epithelium, lung eosinophilia, elevated levels of IgE, accumulation of collagen in submucosa, mast cell hyperplasia, and increased Airway Hyperresponsiveness (AHR), which are characteristics of lungs in human asthmatic patients [60]. Similarly, the systemic expression of IL-9 in transgenic animals resulted in lymphomagenesis, expansion of B-1 lymphocytes, high levels of IgE, and mastocytosis. Similar histopathological changes which are characteristics of human asthma are induced in animals after lung instillation of recombinant IL-9 (rIL-9) for ten days [61]. In a mouse model of Chronic Obstructive Pulmonary Disease (COPD), IL-9 plays a role in aggravating the lung injury by increasing inflammatory and oxidative stress in a STAT3-dependent manner [62]. These studies using IL-9 expressing transgenic animals and rIL-9 revealed that IL-9 plays a pivotal role in the development of airway inflammation, mucus production, airway hyperresponsiveness, and airway fibrosis. 

High levels of IL-9 are also observed in patients with allergic rhinitis and a peanut allergy [63,64]. In the absence of IL-9 producing mast cells, mice failed to develop intestinal mastocytosis and food allergy symptoms [65]. Studies with IL-9 overexpressing mice revealed that IL-9 also plays role in gastrointestinal allergies. In an animal model of allergic asthma, exposure to Aspergillus fumigatus or a dust mite antigen resulted in an allergic inflammatory response in mice including significant increases in Broncho Alveolar lavage (BAL) eosinophils, high serum IgE levels, increased mucin production, and enhanced AHR. The intratracheal administration of an IL-9-neutralizing antibody reduced the allergic inflammation in these animals [66]. Similar results were obtained with the systematic administration of an IL-9 neutralization antibody in ovalbumin-treated BALB/c mice [67]. These observations revealed that IL-9 is a critical player in allergic inflammatory responses.

Promising results in animal studies have directed two randomized placebo-controlled studies to assess the safety profile and potential efficacy of different doses of MEDI-528, a humanized anti-IL-9 monoclonal antibody, in asthma patients [68]. In study I, 36 mild asthma patients (18–65 years), received MEDI-528 (0.3, 1, and 3 mg/kg) or a placebo subcutaneously twice weekly for four weeks. In this study, however, MEDI-528 failed to show any effect on pulmonary function. In study II, nine adults (18–50 years) with stable, mild to moderate asthma and exercise-induced bronchoconstriction (EIB) received 50 mg MEDI-528 or a placebo subcutaneously twice weekly for four weeks. This study indicated that blocking IL-9 with MEDI-528 may affect EIB; however, due to the limited small sample size, statistical analysis was not performed. In order to further investigate whether MEDI-528 has any clinical benefits in patients with asthma, a double blind, multicenter phase IIb study was therefore performed [69]. In the phase II study, 329 adults were enrolled and randomized (1:1:1:1) to subcutaneous placebo control or different doses of MEDI-528 (30, 100, and 300 mg) for 24 weeks. Patients were given MEDI-528 or a placebo every two weeks in addition to the asthma medicines. The study did not show any improvement in the Asthma Control Questionnaire-6 scores, which was the primary endpoint of the study after the addition of MEDI-528 to the existing asthma medications in patients. In summary, although blocking IL-9 showed a promising outcome in the animal model, it failed to show any efficacy in patients with moderate-to-severe asthma. The reason is not clear but may be due to the limitations of animal models of asthma to perfectly mimic the chronic inflammatory response observed in asthmatic patients.

### 5.2. Autoimmune Diseases

Autoimmune diseases include more than 80 chronic diseases among which commonly include multiple sclerosis, inflammatory bowel disease, rheumatoid arthritis, and systemic lupus erythematosus. Over the last few decades, the global prevalence of these diseases has been steadily increasing [70]. Though the aetiology of these diseases remains obscure, it is considered to involve multiple factors such as genetic, environmental, hormonal, and immunological factors that result in skewing the immune response away from immune tolerance and mounting towards harmless self-antigens which ultimately results in inflammation, tissue damage, and loss of function of the affected tissues or organs. 

#### 5.2.1. Lupus Nephritis

In patients with systemic lupus erythematosus (SLE), higher levels of IL-9 have been observed in the serum compared to healthy controls; however, no significant correlation was observed between the IL-9 level and the SLE disease activity index [71,72]. A high expression of IL-9 was also observed in the kidneys and spleens of lupus-prone mice MRL/lpr [73]. Increased IL-9 levels in the serum closely related to the production of antibodies against double-stranded DNA (dsDNA) and positively correlated with serum dsDNA titers in these animals. In MRL/lpr mice, treatment with a neutralizing anti-IL-9 antibody alleviated lupus nephritis and decreased serum titers of anti-dsDNA antibodies. These observations clearly demonstrate that IL-9 is a good therapeutic target for SLE.

#### 5.2.2. Inflammatory Bowel Diseases 

Inflammatory Bowel Diseases (IBDs) such as Crohn’s disease (CD) and ulcerative colitis (UC) are chronic inflammatory disorders of the gastrointestinal tract. Significant increases in the expression of *Il9* mRNA levels can be observed in mucosal biopsies from patients with UC as compared to healthy controls [74]. In addition, in patients with UC, the expression of *Il9* correlated with the activity of the disease, as assessed by endoscopy (Mayo score) [75]. High IL-9-expressing cells are also observed in patients with CD compared with control patients. Similarly, IL-9R is overexpressed on gut epithelial cells in patients with UC and CD. In a mouse model of colitis induced by the hapten oxazolone, expression of *Il9* is upregulated similar to UC. IL-9 deficiency or neutralization protected mice from the development of acute colitis. In *Rag* (Recombination-activating gene) RAG deficient mice, the transfer of IL-9 producing T_H_9 cells resulted in UC, suggesting that T_H_9 cells have a pathogenic functions of in disease progression in IBD. T cell-mediated colitis induced by the hapten reagent 2,4,6-Trinitrobenzenesulfonic acid (TNBS) is a good animal model to study IBD as it shares similarity with Crohn’s disease in humans. IL-9-deficient mice were almost completely protected from TNBS colitis, underlining again that IL-9-mediated signaling plays an important role in T cell-dependent intestinal inflammation [76]. 

The expression of tight junction proteins like claudins and occludin are essential for maintaining the integrity of the intestinal barrier and alterations in their expression is observed in numerous inflammatory disorders. In vivo wound-healing studies demonstrated that administration of recombinant IL-9 impaired intestinal wound healing, while IL-9 deficiency favored wound closure [76]. The effect was associated with the increase in expression of claudin-2 expression with IL-9 treatment and reduction in claudin-2 expression with IL-9-deficiency, indicating that IL-9 disrupts intestinal permeability by enhancing the expression of claudin-2, which helps in promotion of colitis. Contradictory observations were reported in Dextran Sulfate Sodium (DSS)-induced model of colitis mice. In one study, the injection of anti-IL-9 antibody for two weeks reduced the severity of inflammation in DSS-induced colitis mice, suggesting the role of IL-9 in pathogenesis of UC [77]. In another study, IL-9 secreted from invariant natural killer T cells resulted in the resolution of intestinal inflammation through suppression of IFN-γ and IL-17A, as well as the enhancement of IL-10 and TGF-β production [78]. Thus, in IBD, the cellular source of IL-9 determines whether it plays a role in the pathogenesis of the disease or in the resolution of the inflammation. However, detailed animal studies are essential to understand the complex biology of IL-9. Reduced levels of IL-9 are also observed in the serum of patients with CD when infliximab, an antibody directed against TNF-α, was administered, suggesting that IL-9 may be a promising novel biomarker for CD monitoring [79].

#### 5.2.3. Multiple Sclerosis

Multiple Sclerosis (MS) is an autoimmune disorder where T cells specific for myelin protein mediate an inflammatory process that results in demyelination of central nervous system (CNS). Experimental autoimmune encephalomyelitis (EAE) is an animal model of human MS.

Data obtained from various studies in EAE are contradictory regarding the role of IL-9. One report demonstrated that IL-9 neutralization and IL-9R deficiency attenuated the disease, which correlated with a decrease in Th17 cells and IL-6-producing macrophages in the central nervous system (CNS) [41]. The suppression of EAE in IL-9 knock out mice is attributable to the down regulation of IL-17, IFN-γ, TNF-α, IL-12p70, and the inhibition of chemokine receptors C-C chemokine receptor 2 (CCR2), CCR5, and—in particular—CCR6 in activated T cells, which are necessary for the migration of pathogenic T cells into the CNS [80]. Another study, however, showed that mice lacking the IL-9 receptor (IL-9R−/−) exhibited a more severe form of EAE due to the weaker immunosuppressive functions of nTregs resulting in an increase in inflammation [42]. Thus, the role of IL-9 in multiple sclerosis is not clear from the outcome of studies in animal model. Measurement of IL-9 in the cerebrospinal fluid of relapsing remitting (RR) MS patients and healthy individuals revealed no significant differences [81]. Therefore, in vitro functional data from patient samples are necessary to understanding the role of IL-9 in multiple sclerosis.

#### 5.2.4. Myasthenia Gravis

Myasthenia gravis (MG) is one of the rare organ-specific autoimmune diseases in which the neuromuscular transmission is affected is one of the rare organ-specific autoimmune diseases in which the neuromuscular transmission is affected due to the presence of auto-antibodies against the nicotinic acetylcholine receptor (AChR) [82]. Experimental autoimmune myasthenia gravis (EAMG), an animal model of MG in which the immunization of susceptible mouse and rat strains is given on Day 0 with the AChR peptide emulsified in complete Freund’s adjuvant (CFA), followed by booster on day 30 with AChR peptide emulsified in Incomplete Freund’s adjuvant (IFA) [83]. A study by Yao et al. observed an increase in the percentage of T_H_9 cells during the chronic phase of the EAMG [84]. Therefore, the authors checked the effect of neutralizing IL-9 on disease progression. Rats treated with a high dose of anti-IL-9 antibody showed a lower average clinical score and reduced weight loss compared to untreated rats during the chronic phase. Furthermore, the authors observed a decrease in the level of anti-AChR IgG in the sera of animal treated with anti-IL-9 antibodies as the treatment affected B cell differentiation. These data suggest that IL-9 plays an important pathogenic role in EAMG, and that anti-IL-9 antibody treatment might represent a promising therapy for MG. 

#### 5.2.5. Inflammatory Arthritis

In rheumatoid arthritis (RA), an increased expression of IL-9 and IL-9R is observed in synovial tissue, which also correlates with the degree of tissue inflammation [85]. Our group has recently shown that IL-9 prolongs the survival of neutrophils, enhances the production of matrix metalloprotienase-9, and facilitates the differentiation of T_H_17 cells, thus indicating its pathogenic role in RA [86]. However, Rauber et al., showed in an animal model of RA that IL-9-producing type 2 innate lymphoid cells (ILC2s) play a role in resolution of chronic inflammation [87]. Thus, in RA it appears that IL-9 can play a role in pathogenesis as well as in the resolution of chronic inflammation, depending on the cellular source and the existing microenvironment.

In psoriatic patients, increased IL-9R expression is observed in skin lesions. In addition, IL-9 producing T_H_9 cells are increased in the skin lesions [88]. Interestingly, the percentage of Th9 cells in psoriatic patient decreases after anti-TNF and ustekinumab (anti-IL-12/IL-23) treatment suggesting that IL-9 can be a good marker for disease monitoring [89]. IL-9 has been observed to induce T_H_17-dependent psoriasis-like skin inflammation and angiogenesis in K5. hTGF-b1 transgenic mice, suggesting that there is a pathogenic involvement of IL-9 in psoriasis [88].

These observation from animal models and human samples clearly suggest that IL-9 plays a pathogenic role in various inflammatory diseases which are summarized in Table 1 and can be a good therapeutic target for future clinical intervention.

### 5.3. Immune Tolerance

There are few reports which indicate that IL-9 also has role in inducing immune tolerance. In skin allograft transplantation, IL-9 has been shown to be instrumental in maintenance of a tolerant environment [45]. For Treg-cell-dependent allograft tolerance, mast cells are critical player, as mast-cell-deficient mice are not capable of inducing tolerance. The same study showed that activated Treg cells produce high levels of IL-9, which recruit and activate mast cells, a process essential for inducing tolerance. Therefore, IL-9 neutralization accelerated the rejection of allograft in tolerant Rag –/– mice [90]. Another study compared the serum levels of IL-9 between healthy controls and stable liver transplant recipients who were free of rejection episodes for at least eight years [91]. Significantly higher concentrations of IL-9 were observed in liver transplant recipients compared to healthy subjects. In addition, patients with low blood level of calcineurin inhibitors (CNI) showed higher serum levels of IL-9, indicating that IL-9 may play a role in induction of tolerance in liver transplantation as well.

## 6. Outlook

Though IL-9 has been underrated for many years, the recent attention in IL-9 biology has provided interesting aspects of its cellular sources and targets in pathogenic as well as physiological conditions. In the last few decades, multiple signaling pathways and transcription factors have been identified which regulate the expression of IL-9. However, the lineage or cell specific transcription factor remains elusive till date. Most of the signaling intermediates of IL-9/IL-9R pathway have been identified in the cell lines, and future attempts must be directed towards understanding the IL-9/IL-9R signaling and its regulation in pathological conditions. In addition, limited information is available about the soluble form of IL-9R in diseased condition. A study in Japanese individuals revealed high levels of soluble IL-9R in patients with diarrhea positive hemolytic uremic syndrome compared to age matched healthy controls [92]. Thus, it would be interesting to study the level of soluble IL-9R in various diseases and to understand its relation with the disease progression.

The emerging role of IL-9 on inflammation, resolution, and tolerance suggest that targeting the IL-9 pathway can be considered a potential strategy for treating different kinds of inflammatory diseases. The studies in animal models of inflammatory diseases have reflected the influence of the cellular origin in determining the role of IL-9 in inflammation or resolution. Therefore, extensive future studies are necessary to better understand the regulation of IL-9 expression in various cell types and the cell specific IL-9 signaling pathway to dissect out the molecular pathway for its anti-inflammatory and pro-inflammatory functions.

## Figures and Tables

**Table 1:** Status of IL-9 in various inflammatory diseases.

Inflammatory Conditions	Expression of IL-9 or IL-9R on Patient Samples	Animal Studies	References
Asthma and Allergy	Increased IL-9 levels in lungs, sputum and sera of asthmatic patients. Elevated expression of IL-9R in the lungs of asthmatic patients. High levels of IL-9 are also observed in patients with allergic rhinitis and peanut allergy.	Overexpression of IL-9 in the lungs of transgenic mice, systemic expression of IL-9 in transgenic animal, and instillation of recombinant IL-9 in the lungs of animals resulted in histopathological changes of lung characteristic of human asthma. Neutralizing IL-9 reduced the allergic inflammation in animal models	[55,56,57,58,59,60,61,63,64,65]
COPD	-	IL-9 aggravates the lung injury in a mouse model of COPD by increasing inflammatory and oxidative stress in a STAT3 dependent manner.	[62]
Systemic lupus erythematosus (SLE)	Higher levels of IL-9 in the serum of SLE patient compared to healthy controls.	In MRL/lpr mice treatment with a neutralizing anti-IL-9 antibody alleviated lupus nephritis.	[71,72,73]
Inflammatory bowel diseases (IBDs)	Higher expression of *Il9* in mucosal biopsies from patients with ulcerative colitis (UC) as compared to healthy controls. In patients with UC, expression of *Il9* mRNA correlated with the activity of the disease, as assessed by endoscopy (Mayo score) More IL-9-expressing cells were observed in patients with Crohn’s disease (CD) than in control patients. IL-9R was overexpressed on gut epithelial cells in patients with UC or CD	IL-9 deficiency or neutralization protected mice from the development of acute colitis. IL-9-deficient mice were almost completely protected from TNBS induced colitis model. Contradictory reports in DSS induced colitis animal model. In one study, anti-IL-9 antibody injection for two weeks reduced the severity of inflammation in DSS induced colitis mice. In another study, IL-9 secreted from invariant natural killer T cells resulted in resolution in intestinal inflammation through suppression of IFN-γ and IL-17A, but enhancement of IL-10 and TGF-β.	[74,75,76,77,78]
Multiple Sclerosis	No significant difference in the IL-9 level in the cerebrospinal fluid of relapsing remitting (RR) MS patients compared to healthy individuals	Contradictory observations, in one study IL-9 neutralization and IL-9R deficiency attenuated the disease. In another study, IL-9R KO mice exhibited a more severe course of experimental autoimmune encephalomyelitis (EAE).	[41,42,80,81]
Myasthenia gravis	-	Neutralization of IL-9 improved disease in experimental autoimmune myasthenia gravis (EAMG).	[84]
Rheumatoid Arthritis (RA)	In RA patients, increased expression of IL-9 and IL-9R is observed in the synovial tissue, which correlates with the degree of tissue inflammation	In animal model of RA, IL-9-producing type 2 innate lymphoid cells (ILC2s) play a role in resolution of chronic inflammation.	[85,86,87]
Psoriasis	Increased expression of IL-9R and high frequency of Th9 is observed in skin lesions of patient	In an animal model of psoriasis, IL-9 promotes skin inflammation.	[88,89]

## Abbreviations

AHR	Airway Hyperresponsiveness
BAL	Broncho Alveolar Lavage
CCR	C-C Chemokine Receptor
CD	Crohn’s Disease
CIS	Cytokine-Inducible SH2-Containing Protein
COPD	Chronic Obstructive Pulmonary Disease
DSS	Dextran Sulfate Sodium
EAE	Experimental Autoimmune Encephalomyelitis
EIB	Exercise-Induced Bronchoconstriction
IRS	Insulin Receptor Substrate
IBD	Inflammatory Bowel Diseases
JAK	Janus Kinases
MEA	Mast Cell Growth Enhancing Activity
MG	Myasthenia Gravis
MS	Multiple Sclerosis
NKT	Natural Killer T Cells
PDK	PI-3K Dependent KINASE
PI-3K	Phosphatidylinositol-3 KINASE
PIAS	Protein Inhibitors of Activated STATs
PTB	Protein Tyrosine Binding
RA	Rheumatoid Arthritis
RR	Relapsing Remitting (RR)
SH2	Src Homology 2
SOCS	Suppressors Of Cytokine Signaling
SLE	Systemic Lupus Erythematous
STAT	Signal Transducer and Activator of Transcription
TCGF	T Cell Growth Factor
TNBS	Tri Nitro Benzene Sulfonic
UC	Ulcerative Colitis
